# Activation of NAG-1 via JNK signaling revealed an isochaihulactone-triggered cell death in human LNCaP prostate cancer cells

**DOI:** 10.1186/1471-2407-11-146

**Published:** 2011-04-20

**Authors:** Sheng-Chun Chiu, Mei-Jen Wang, Hsueh-Hui Yang, Shee-Ping Chen, Sung-Ying Huang, Yi-Lin Chen, Shinn-Zong Lin, Horng-Jyh Harn, Cheng-Yoong Pang

**Affiliations:** 1 Institute of Medical Sciences, Tzu-Chi University, Hualien, Taiwan; 2 Department of Medical Research, Buddhist Tzu-Chi General Hospital, Hualien, Taiwan; 3 Tzu-Chi Stem Cell Centre, Buddhist Tzu-Chi General Hospital, Hualien, Taiwan; 4 Department of Ophthalmology, Mackay Memorial Hospital, Hsinchu, Taiwan; 5 Graduate Institute of Biotechnology, National Ilan University, Ilan, Taiwan; 6 Center for Neuropsychiatry, China Medical University Hospital, Taichung, Taiwan; 7 Pathology Department, China Medical University, Taichung, Taiwan

## Abstract

**Background:**

We explored the mechanisms of cell death induced by isochaihulactone treatment in LNCaP cells.

**Methods:**

LNCaP cells were treated with isochaihulactone and growth inhibition was assessed. Cell cycle profiles after isochaihulactone treatment were determined by flow cytometry. Expression levels of cell cycle regulatory proteins, caspase 9, caspase 3, and PARP were determined after isochaihulactone treatment. Signaling pathway was verified by inhibitors pre-treatment. Expression levels of early growth response gene 1 (EGR-1) and nonsteroidal anti-inflammatory drug-activated gene 1 (NAG-1) were determined to investigate their role in LNCaP cell death. NAG-1 expression was knocked down by si-NAG-1 siRNA transfection. Rate of cell death and proliferation were obtained by MTT assay.

**Results:**

Isochaihulactone caused cell cycle arrest at G2/M phase in LNCaP cells, which was correlated with an increase of p53 and p21 levels and downregulation of the checkpoint proteins cdc25c, cyclin B1, and cdc2. Bcl-2 phosphorylation and caspase activation were also observed. Isochaihulactone induced phosphorylation of c-Jun-N-terminal kinase (JNK), and JNK inhibitor partially reduced isochaihulactone-induced cell death. Isochaihulactone also induced the expressions of EGR-1 and NAG-1. Expression of NAG-1 was reduced by JNK inhibitor, and knocking down of NAG-1 inhibited isochaihulactone-induced cell death.

**Conclusions:**

Isochaihulactone apparently induces G2/M cell cycle arrest via downregulation of cyclin B1 and cdc2, and induces cellular death by upregulation of NAG-1 via JNK activation in LNCaP cells.

## Background

Prostate cancer is the most common malignancy in American men and the second leading cause of deaths from cancer [1]. In the early stage, prostate cancer usually grows slowly and remains confined to the gland, initially producing few or no symptoms. As the cancer advances, it can, however, spread beyond the prostate into the surrounding tissues and to other areas, such as the bones, lungs, and liver. Therefore, symptoms often appear after the cancer has processed to an advanced stage.

The treatment options for patients with prostate cancer include surgery, radiation therapy, hormonal therapy, chemotherapy, cryotherapy, and combinations of some of these treatments. At the early stage, surgery, radiation therapy, and hormonal therapy are the preferred treatments. As the cancer processes, chemotherapy and cryotherapy become the preferred treatments. One of the most common drug classes for chemotherapy treatments for prostate cancer is the taxanes, which include the first-generation drug paclitaxel (Taxol, a trademark of Bristol-Myers Squibb) [2,3]. Because taxanes often cause significant negative side effects, newly developed drugs are valuable.

Recently, non-traditional treatments such as herbs and dietary supplements have been considered as alternative medicines. Nan-Chai-Hu (Chai Hu of the South), the root of *Bupleurum scorzonerifolium*, is an important Chinese herb in the treatment of influenza, fever, malaria, cancer, and menstrual disorders in China, Japan, and many other parts of Asia. We previously showed that the crude acetone extract of *B. scorzonerifolium *(BS-AE) causes cell cycle arrest at the G2/M phase and apoptosis in the human lung carcinoma cell line A549 [4-6]. After the acetone extract fraction was further purified, a novel lignan, isochaihulactone, which has antitumor activity against A549 cells *in vitro *and *in vivo*, was identified [7]. Isochaihulactone induces G2/M arrest and apoptosis in cancer cells. This compound can also be isolated from *Bursera microphylla *(Burseraceae) and shows antitumor effects [8].

Here we describe the anti-tumor activity of isochaihulactone, which causes cell cycle arrest at G2/M phase and cell death in LNCaP cells. We provided evidence that the disruption of the cell cycle at G2/M phase and the activation of phospho-Bcl-2 and caspase-3 are important in isochaihulactone-induced cell death. Recently, we found isochaihulactone induces growth inhibition and apoptosis in A549 cells by activating early growth response gene 1 (EGR-1) and nonsteroidal anti-inflammatory drug-activated gene 1 (NAG-1) through an extracellular signal-regulated kinase 1/2 (ERK 1/2)-dependent pathway, but PI3K signaling is not involved [9]. Here we show that isochaihulactone induced growth inhibition and cell death in prostate cancer cells by activating EGR-1 and NAG-1 through JNK-dependent pathway and that did not involve activation of ERK signaling. Also, isochaihulactone-induced cell death can be restored by siNAG-1 siRNA transfection. Our findings indicate that isochaihulactone is a potential antitumor compound for prostate cancer therapy.

## Methods

### Cells and cell culture

LNCaP human prostate cells, obtained from ATCC (American Type Culture Collection, Manassas, VA), were cultured in RPMI 1640 medium with 10% heat-inactivated fetal bovine serum, 100 U/ml penicillin and 100 U/ml streptomycin, 1% sodium pyruvate, 2 mM L-glutamine (all of these reagents are from Invitrogen, Carlsbad, CA) at 37°C in a humidified atmosphere with 5% CO_2_. Cells were plated in 6-well plates at a seeding density of approximately 2 × 10^5 ^cells/well in the presence or absence of isochaihulactone (20 μM).

### Chemicals and reagents

*Bupleurum scorzonerifolium *roots were supplied by Chung-Yuan Co. (Taipei, Taiwan). The plant was identified and deposited at National Defense Medicinal Center (NDMCP No. 900801). Isochaihulactone (4-benzo[1,3]dioxol-5-ylmethyl-3-(3,4,5-trimethoxyl-benzylidene)-dihydro-furan-2-one) was prepared as described previously [7]. RPMI 1640 medium, fetal bovine serum (FBS), penicillin, streptomycin, L-glutamine, sodium pyruvate, trypsin/EDTA were purchased from Invitrogen. The RNA isolation kit was purchased from QIAGEN (Valencia, CA). Dimethyl sulfoxide (DMSO), 3-(4,5-dimethyl thizol-2-yl)-2,5-diphenyl tetrazolium bromide (MTT), paclitaxel, and horseradish peroxidase-conjugated secondary antibodies were purchased from Sigma Chemical Co. (St. Louis, MO, USA). The ERK1/2 kinase inhibitor PD98059 and the JNK inhibitor SP600125 were purchased from R&D Systems (Minneapolis, MN). The p38 inhibitor SB203580 and the PI3K/AKT inhibitor LY294002 were purchased from Calbiochem (San Diego, CA). The annexin-V-FLUOS Staining Kit was from Roche Molecular Biochemicals (Mannheim, Germany). Polyvinyldenefluoride (PVDF) membranes, BSA protein assay kit and western blot chemiluminescence reagent were purchased from Amersham Biosciences (Arlington Heights, IL).

### Western blot analysis

LNCaP cells were lysed on ice with 200 μl of lysis buffer (50 mM Tris-HCl, pH 7.5, 0.5 M NaCl, 5 mM MgCl2, 0.5% Nonidet P-40, 1 mM phenylmethylsulfonyl fluoridefor, 1 μg/ml pepstatin, and 50 μg/ml leupeptin) and centrifuged at 13,000 × g at 4°C for 5 min. The protein concentrations in the supernatants were quantified using a BSA Protein Assay Kit. Electrophoresis was performed on a NuPAGE Bis-Tris Electrophoresis System using 30 μg of reduced protein extract per lane. Resolved proteins were then transferred to PVDF membranes. Membranes were blocked with 5% non-fat milk for 1 h at room temperature and probed with appropriately dilution of primary antibodies at 4°C overnight: NAG-1/PTGF-b (1:1000, Upstate Biotechnology, Lake Placid, NY), phospho-ERK1/2 (1:2000), ERK1/2 (1:2000), phospho-p38 (1:1000), p38 (1:1000), phospho-JNK1/2 (1:1000), JNK1/2 (1:1000), cyclin B1 (1:1000), cdc2 (1:1000), cleaved Caspase-3 (Asp175) (1:1000), cleaved Caspase-8 (1:1000), cleaved Caspase-9 (Asp330) (1:1000), PARP (46D11) (1:1000), phospho-Bcl-2 (ser70) (1:1000), p53 (1:1000), were purchased from Cell Signaling Technology, Inc. (Danvers, MA). After the PVDF membrane was washed three times with TBS/0.2% Tween 20 at room temperature, it was incubated with appropriate secondary antibody (goat anti-mouse or anti-rabbit, 1:10000, Sigma Chemical, St. Louis, MO) labeled with horseradish peroxidase for 1 h at room temperature. All proteins were detected using Western Lightning™ Chemiluminescence Reagent Plus (Amersham Biosciences, Arlington Heights, IL) and quantified with densitometers.

### Growth inhibition assay

The viability of the cells after treatment with various chemicals was evaluated using MTT assay preformed in triplicate. Briefly, the LNCaP cells (2 × 10^5^/well) were incubated in 6-well plates containing 2 ml of serum-containing medium. Cells were allowed to adhere for 18-24 h and then were washed with phosphate-buffered saline (PBS). Solutions were always prepared fresh by dissolving 0.2% DMSO (control) or drugs in culture medium before their addition to LNCaP cells. For inhibitor treatment experiments, cells were pre-incubated for 1 h with 25 μM and 50 μM ERK1/2 kinase inhibitor PD98059, 10 μM and 20 μM p38k inhibitor SB203580, or 10 μM and 20 μM JNK inhibitor SP600125 and then were treated with 20 μM isochaihulactone for 24 h. The drug-containing medium was removed, cells were washed with PBS, and culture medium containing 300 μg/ml MTT was added for 1 h at 37°C. After the medium were removed, 2 ml of DMSO were added to each well. Absorbance at 570 nm of the maximum was detected by a PowerWave × Microplate ELISA Reader (Bio-Tek Instruments, Winooski, VT). The absorbance for DMSO-treated cells was considered as 100%. The results were determined by three independent experiments.

### Cell cycle analysis

The cell cycle was determined by flow cytometry following DNA staining to reveal the total amount of DNA. Approximately 5 × 10^5 ^of LNCaP cells were incubated with 20 μM isochaihulactone for the indicated time. Cells were harvested with trypsin/EDTA, collected, washed with PBS, fixed with cold 100% ethanol overnight, and then stained with a solution containing 45 mg/ml PI, 10 mg/ml RNase A, and 0.1% Triton X-100 for 1 h in the dark. The cells were then passed through FACScan flow cytometer (equipped with a 488-nm argon laser) to measure the DNA content. The data were obtained and analyzed with CellQuest 3.0.1 (Becton Dickinson, Franklin Lakes, NJ) and ModFitLT V2.0 software.

### Transfection with siRNA

NAG-1 siRNA was designed by siGENOME SMARTpool duplex siRNA and purchased from Dharmacon RNAi Technologies (Chicago, IL). LNCaP cells at 50 to 60% confluence were transfected with NAG-1 siRNA (10-50 nM) for 48 h using RNAifect Transfection Reagent (QIAGEN). The medium was removed, and the cells were treated with isochaihulactone or vehicle for up to 48 h. Proteins were then isolated for western blotting, or cells were collected for the MTT assay.

### Immunocytochemistry

LNCaP cells cultured on glass slides were treated with 20 μM isochaihulactone for 48 h prior to fixation with cold 4% paraformaldehyde. The fixed cells were washed twice in PBS, and incubated in cold permeabilization solution (0.3% Triton X-100 + 0.1% sodium citrate). After endogenous peroxidase activity was inactivated with 3% H_2_O_2_, the cells were washed with PBS and incubated with an anti-cleaved caspase-3 at 4°C overnight. The cells were washed with PBS three times and then incubated with FITC-conjugated secondary antibody 1 h at room temperature. The cells were then washed with PBS three times and stained with 300 nM DAPI for 10 min. Images were obtained with a confocal microscope (Carl Zeiss, Oberkochen, Germany).

### TUNEL assay

LNCaP cells were cultured in the presence or absence of isochaihulactone (20 μM) for 60 h and then examined for apoptosis with TUNEL assay (*In Situ *Cell Death Detection Kit, Roche).

### Statistical analysis

The data are shown as mean ± S.D. Statistical differences were analyzed using the Student's *t*-test for normally distributed values and by nonparametric Mann-Whitney *U*-test for values with a non-normal distribution. Values of *P *< 0.05 were considered significant.

## Results

### Isochaihulactone inhibited proliferation and induced morphology changes of the human prostate cancer cells

**Figure 1:**
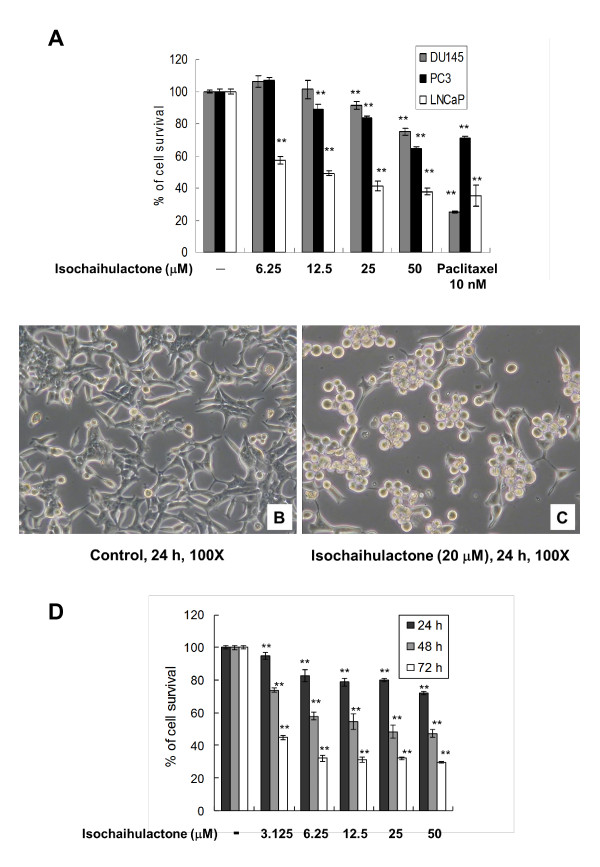
**Morphological changes and anti-proliferation effects after isochaihulactone treatment of prostate cancer cells**. (**A**) Human prostate cancer cell lines DU-145, PC-3, LNCaP were treated with isochaihulactone from 6.25 to 50 μM at 48 h and analyzed with the MTT assay. LNCaP cells were treated with 0.2% DMSO as a control (**B**) or 20 μM isochaihulactone (**C**) for 24 h. LNCaP cells were treated with increasing concentration of isochaihulactone from 3.125 to 50 μM at various times from 24 to 72 h and analyzed with the MTT assay (**D**). The data represent the means ± S.D. from three independent experiments. **, *P *< 0.01 versus vehicle.

Isochaihulactone has a strong anti-proliferative effect on A549 cells and caused G2/M phase arrest and apoptosis in a time- and concentration-dependent manner [7]. To determine the cytotoxicity of isochaihulactone on prostate cancer cells, three human prostate cancer cell lines, namely, DU-145, PC3, and LNCaP were tested. The MTT assay revealed that isochaihulactone had a strong anti-proliferative effect on human prostate cancer cell lines, especially the LNCaP cells (Figure 1A). LNCaP cells were selected for subsequent studies. Compared with untreated cells, isochaihulactone-treated LNCaP cells showed obvious cell shrinkage and rounding up, features typical of cells undergoing apoptosis (Figure 1B and 1C). The MTT assay showed that isochaihulactone had anti-proliferative effects on LNCaP cells that were time- and dose-dependent (Figure 1D). Treatment of LNCaP cells with 25 μM isochaihulactone for 48 h resulted in 48.3% cell survival, whereas treatment for 72 h resulted in 32% cell survival (Figure 1D). Based on these data, we used 20 μM isochaihulactone for subsequent studies (50.5% cell survival after 48hr treatment and data not shown).

### Isochaihulactone induced cell cycle arrest in G2/M phase and changed the expression levels of G2/M regulatory proteins

**Figure 2:**
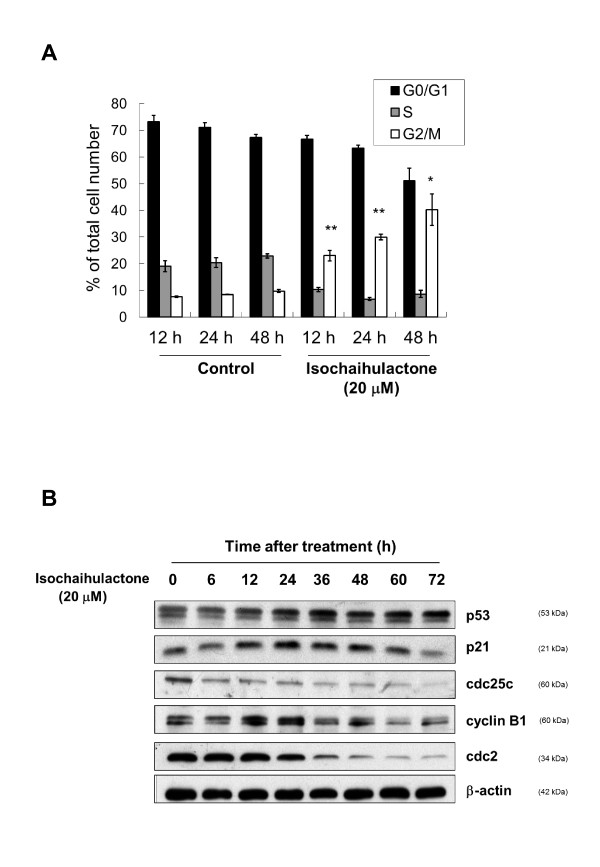
**Isochaihulactone apparently induces G2/M phase arrest and changes the expression profiles of G2/M regulatory proteins**. (**A**) Isochaihulactone induced cell cycle arrest at G2/M in LNCaP cells. For the cell cycle analysis, cells were seeded at 5 × 10^5 ^per 5-cm plate in triplicates and treated with 20 μM isochaihulactone for 12-48 h. The data represent the means ± S.D. from three different experiments. *, *P *< 0.05; **, *P *< 0.01; versus control. (**B**) Cells were treated with 20 μM isochaihulactone for 6-72 h. Western blot analysis of p53, p21, cdc25c, cyclin B1 and cdc2 was performed. β-actin was used as an internal control.

In order to elucidate its mode of action, we examined effects of isochaihulactone on cell cycle progression. Flow cytometry analysis showed that isochaihulactone treatment resulted in the accumulation of cells in G2/M phase in a time-dependent manner (Figure 2A). Quantification of proliferating untreated LNCaP cells showed that 67.3% of cells were in the G0/G1 phase, 22.8% of cells were in the S phase, and 9.7% of cells were in the G2/M phase of cell cycle 48 h after plating. Treatment of LNCaP cells with 20 μM isochaihulactone for 48 h increased the percentage of cells in the G2/M phase to 40.2% and reduced the percentage of the cells in the G0/G1 and S phase (51.1 and 8.6%, respectively). The subdiploid population of cells accounted for ~2%.

To determine the relationship between isochaihulactone-induced mitotic arrest and p53, p21, cdc25c, and cyclinB1/cdc2 activities and Bcl-2 phosphorylation, we first examined the expression of these G2/M regulatory proteins in LNCaP cells treated with 20 μM isochaihulactone for increasing times. Western blot analysis showed that treatment of LNCaP cells with isochaihulactone resulted in upregulation of p53 and p21 and downregulation of cdc25c, cyclin B1, and cdc2 in a time-dependent manner (Figure 2B). These data suggest that isochaihulactone apparently induced LNCaP cells to undergo G2/M growth arrest by affecting the expression of G2/M regulatory proteins.

### Isochaihulactone induced LNCaP cell death

**Figure 3:**
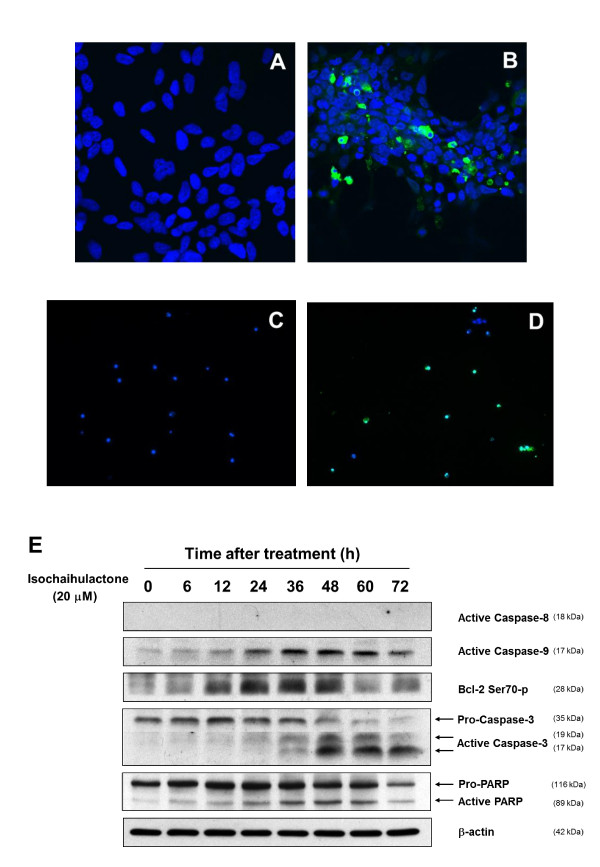
**Isochaihulactone induces cell death and initiates Bcl-2 phosphorylation and caspase activation in LNCaP cells**. LNCaP cells were treated with 0.2% DMSO (**A**) or 20 μM isochaihulactone (**B**) for 48 h and then were fixed and stained for cleaved caspase-3. Nuclei were stained with DAPI. LNCaP cells were treated with 0.2% DMSO (**C**) or 20 μM isochaihulactone (**D**) for 60 h and then were fixed and stained with the TUNEL assay. Nuclei were stained with DAPI. (**E**) Isochaihulactone induced caspase-9 activation, followed by Bcl-2 phosphorylation and then caspase-3 activation. Cells were treated with 20 μM isochaihulactone for the indicated time and analysis by Western blotting. Membranes were probed with caspase-8, phosphor-Asp330 caspase-9, phosphor-Ser70 Bcl-2, cleaved-βaspase-3, PARP antibodies. β-actin was used as an internal control.

To evaluate the role of apoptosis in isochaihulactone-induced cell death, caspase-3 staining and TUNEL staining were performed. After treatment with 20 μM isochaihulactone for 48 h, the LNCaP cells were fixed and stained with anti-caspase 3, an increased number of FITC-positive cells were seen (Figure 3B) as compared to control cells (Figure 3A). To observe the late stage of apoptosis, LNCaP cells treated with 20 μM isochaihulactone for 60 h was collected and stained with TUNEL staining kit. Most of the isochaihulactone-treated cells were TUNEL positive (Figure 3D) as compared with untreated cells (Figure 3C). Because activation of the caspases and cleavage of PARP are crucial mechanisms for induction of apoptosis, their involvement in isochaihulactone-induced cell death was investigated in LNCaP cells. In addition, Bcl-2, which is located on the outer mitochondrial membrane, is important for the suppression of mitochondrial manifestations of apoptosis [10]. We examined whether isochaihulactone-induced cell death was associated with Bcl-2 phosphorylation. Caspase-9 and caspase-3, but not caspase-8, were activated after isochaihulactone treatment (Figure 3E). Thus, isochaihulactone-induced cell death is mediated through a caspase-dependent pathway. We also observed that caspase-9 activation, Bcl-2 phosphorylation, and cleavage of caspase-3 and PARP in a time-dependent manner (Figure 3E).

### Isochaihulactone-induced JNK1/2 activation was followed by growth inhibition of LNCaP cells

**Figure 4:**
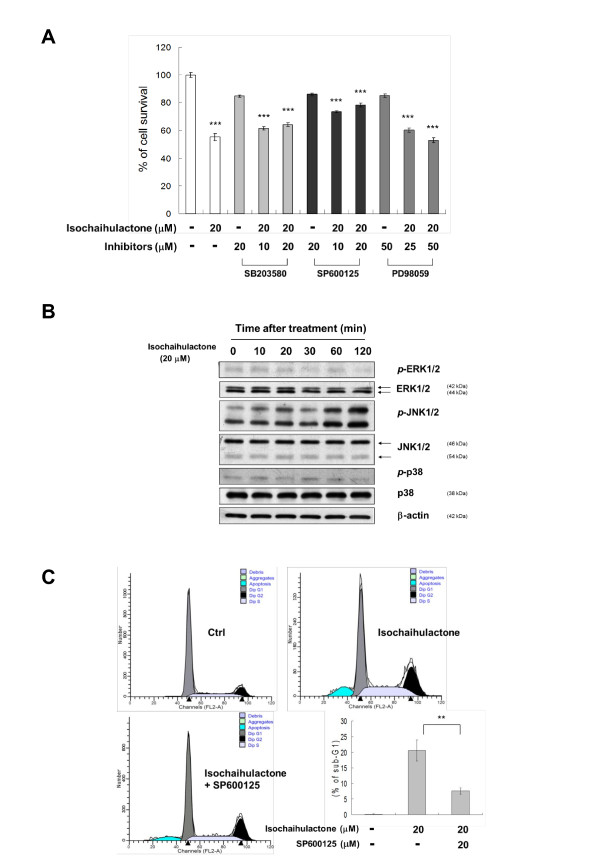
**Growth inhibition of LNCaP cells induced by isochaihulactone is partially rescued by JNK1/2 inhibitor**. (**A**) MTT assay of LNCaP cells pretreated with p38 inhibitor SB203580 (10 or 20 μM), the JNK1/2 inhibitor SP600125 (10 or 20 μM) or the ERK1/2 inhibitor PD98059 (25 or 50 μM) for 1 h and then treated with 20 μM of isochaihulactone for 48 h. The values are the mean ± S.D. from three independent experiments performed in duplicate. (**B**) Cells were treated with 20 μM isochaihulactone for the indicated times. Phospho-ERK1/2, total-ERK1/2, phospho-JNK, total-JNK, phospho-38, total-p38 were detected by western blotting. (**C**) Cells were treated with 20 μM isochaihulactone for 48 h in the presence or absence of JNK1/2 inhibitor SP600125 (20 μM). Cell cycle analysis was done as described in *Methods*. Isochaihulactone-induced sub-G1 population (20.51%) was decreased by JNK1/2 inhibitor SP600125 pre-treatment (7.54%). The data represent the means ± S.D. from three independent experiments. **, P <0.01; ***, *P *< 0.001 versus vehicle.

In our previous study, the anti-proliferative activity of isochaihulactone in A549 cells was via ERK1/2, mitogen-activated protein kinase (MAPK) pathway. To examine whether this pathway is activated in isochaihulactone-treated LNCaP cells, cells were treated with isochaihulactone for 48 h in the presence and absence of the MEK1/2 inhibitor PD98059 (25 or 50 μM), the p38 inhibitor SB203580 (10 or 20 μM), or the JNK1/2 inhibitor SP600125 (10 or 20 μM). Only SP600125 significantly blocked isochaihulactone-induced growth inhibition in a concentration-dependent manner (Figure 4A). We also found that isochaihulactone had no effect on the activation of ERK1/2 (Figure 4B) or PKC (data not shown). Furthermore, to determine which JNK pathways were involved, we evaluated the effect of isochaihulactone on ERK1/2, p38, and JNK1/2 activation. We found that only JNK1/2 showed increased phosphorylation after exposure of LNCaP cells to isochaihulactone for 10-120 min (Figure 4B). In contrast, isochaihulactone had no effect on the phosphorylation of p38 or ERK1/2. To further clarify the role of JNK signaling pathway in isochaihulactone-induced LNCaP cell death, cell cycle analysis was performed in the presence or absence of JNK inhibitor SP600125 by flow cytometry. As shown in Figure 4C, the JNK inhibitor SP600125 (20 μM) significantly reduced the sub-G1 population induced by isochaihulactone from 20.51% to 7.54%. These data suggested that JNK signaling pathway was involved in the mechanism of isochaihulactone-induced cell death.

### Isochaihulactone induced EGR-1 and NAG-1 expression in LNCaP cells

**Figure 5:**
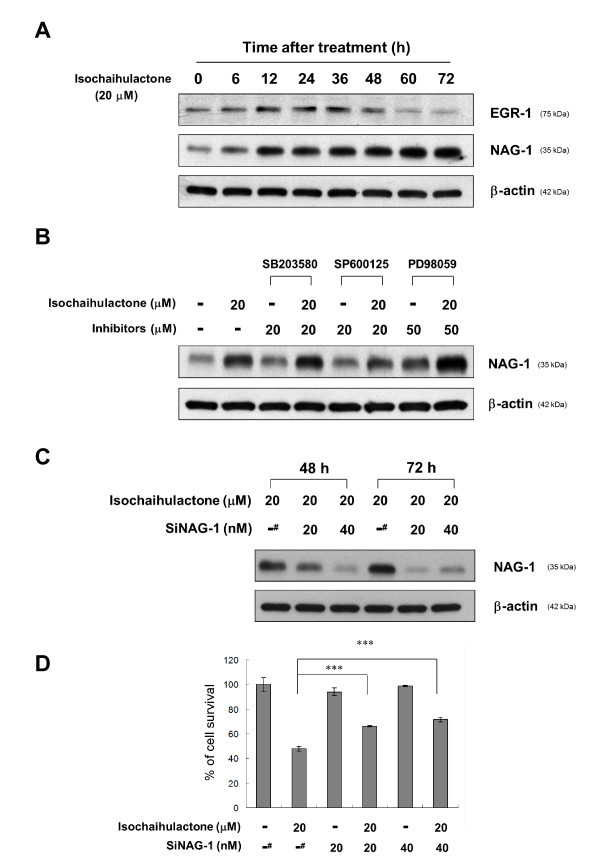
**Isochaihulactone induces NAG-1 expression via JNK1/2 activation, and isochaihulactone-induced cell death can be rescued by NAG-1 siRNA transfection**. (**A**) Expression of EGR-1 and NAG-1 after treatment of LNCaP cells with 20 μM isochaihulactone for the indicated times. (**B**) NAG-1 expression of LNCaP cells pretreated with the p38 inhibitor SB203580 (20 μM), the JNK1/2 inhibitor SP600125 (20 μM), or the MEK1/2 inhibitor PD98059 (50 μM) for 1 h and then treated with 20 μM isochaihulactone for 24 h. (**C**) Suppression of isochaihulactone-induced NAG-1 expression in LNCaP cells by NAG-1 siRNA transfection. LNCaP cells were transfected with scramble siRNA (^#^) or 20 nM, 40 nM NAG-1 siRNA for 48 h using the RNAifect transfection reagent followed by treatment with 20 μM isochaihulactone for 48 or 72 h. Western blot analysis was performed for NAG-1. (**D**) Isochaihulactone-induced anti-proliferative activity was measured with the MTT assay in LNCaP cells transfected with scramble (^#^) or NAG-1 siRNA for 48 h and then treated with 20 μM isochaihulactone for 48 h. The data represent the means ± S.D. from three independent experiments. ***, *P *< 0.001 versus vehicle.

Recently, isochaihulactone was shown to upregulate NAG-1 expression in the human lung carcinoma cell line A549 through an ERK-dependent pathway involving the activation of EGR-1 [9]. To evaluate whether EGR-1 and NAG-1 were involved in the anti-proliferative effect of isochaihulactone in LNCaP cells, the expression of EGR-1 and NAG-1 proteins was determined by western blot analysis. After exposure of cells to isochaihulactone, the expressions of both EGR-1 and NAG-1 were upregulated in a time-dependent manner. EGR-1 was significantly induced at 6 h after isochaihulactone treatment, and this effect was maintained until 36 h. NAG-1 expression occurred later, with the highest expression at 60-72 h (Figure 5A).

### The JNK1/2 signaling pathway was involved in isochaihulactone-induced NAG-1 expression

To investigate a possible role for JNK1/2 in the regulation of NAG-1 expression, LNCaP cells were treated with isochaihulactone (20 μM) in the presence and absence of the p38 inhibitor SB203580 (20 μM), the JNK1/2 inhibitor SP600125 (20 μM), or the MEK1/2 inhibitor PD98059 (50 μM). Using western blot analysis, we found that inhibition of JNK1/2 expression with SP600125 reduced NAG-1 protein levels after treatment of LNCaP cells with isochaihulactone (Figure 5B). In contrast, inhibition of ERK1/2 or p38 had no effect on the induction of NAG-1 (Figure 5B). These results suggest that activation of the JNK1/2 signaling pathway was involved in isochaihulactone-induced NAG-1 expression.

### Induction of NAG-1 was involved in isochaihulactone-induced LNCaP cell death

Since the expressions of EGR-1 and NAG-1 were observed in isochaihulactone-induced A549 apoptotic cell death, their roles in LNCaP cell death were investigated. To determine the role of NAG-1 in the anticancer potential of isochaihulactone in prostate cancer, we used an siRNA approach. Western blot analysis confirmed the suppression of NAG-1 by NAG-1 siRNA in a concentration-dependent manner (Figure 5C). To further characterize the role of NAG-1 in isochaihulactone-induced growth inhibition, LNCaP cells were transfected with siNAG-1 siRNA for 48 h. Then, the MTT assay was performed to determine the percentage of cell death 48 h after treatment with 20 μM isochaihulactone. Nineteen and 24% of cell death was inhibited by 20 and 40 nM NAG-1 siRNA, respectively, after exposure of cells to 20 μM isochaihulactone (Figure 5D). Thus, isochaihulactone-induced cell death in LNCaP cells occurred partially through NAG-1 activation.

## Discussion

In our previous study, we demonstrated that isochaihulactone was efficacious against various models of human solid tumors but not prostate cancer [7]. We also have shown recently that isochaihulactone triggers an apoptotic pathway in human A549 lung cancer cells that occurs via the ERK1/2 and NAG-1 pathway [9]. To clarify the mechanisms of isochaihulactone-induced tumor apoptosis between different types of cancer cells, we further investigated the antitumor potential and mechanisms of isochaihulactone action in human prostate cancer cells. Three human prostate cell lines were used to test the cytotoxicity of isochaihulactone, only the LNCaP prostate cancer cells showed sensitivity to isochaihulactone treatment. This phenomenon might be important to the antitumor potential of isochaihulactone and is discussed later.

In this study, we demonstrated that isochaihulactone apparently induced G2/M cell cycle arrest and cell death in LNCaP cells. The tumor suppressor protein p53 plays a role in the molecular response to DNA damage and cell cycle arrest. The cyclin-dependent kinase inhibitor p21 also helps to maintain G2/M cell cycle arrest by inactivating the cyclin B1/cdc2 complex, disrupting the interaction between proliferating cell nuclear antigen and cdc25c [11]. Our result showed that increased levels of p53 and p21 proteins were expressed in LNCaP cells in response to treatment with isochaihulactone (Figure 2B). The transition from G2 phase to mitosis is triggered by the cdc25c-mediated activation of the cyclin B1/cdc2 complex. Cyclin B1/cdc2 activation is triggered when cdc25c dephosphorylates Thr15 [12,13]. In our study, isochaihulactone-mediated LNCaP cell cycle arrest at G2/M phase (Figure 2B) was accompanied by decreased expression of cyclin B1 and cdc2 kinase. The decrease in the levels of cdc2 may be due to the decrease in cdc25 activation by phosphorylation, leading to subsequent G2 arrest (Figure 2B).

Activation of aspartate-specific cysteine protease (caspase) represents a crucial step in the induction of drug-induced apoptosis, and cleavage of PARP by caspase-3 is considered to be one of the hallmarks of apoptosis [14]. Isochaihulactone-induced caspase 3 cleavage was observed by immunocytochemistry (Figure 3B), and late-stage apoptosis was revealed by TUNEL staining (Figure 3D). Furthermore, isochaihulactone inhibited Bcl-2 expression, induced caspase-9 and caspase-3 cleavage, and induced PARP activation were also observed (Figure 3E). It is interesting to note that isochaihulactone-induced Bcl-2 phosphorylation, caspase-9 cleavage, and PARP cleavage were observed at nearly the same time point, suggesting that the isochaihulactone-induced Bcl-2 phosphorylation is related apoptosis (Figure 3E). Recent reports have revealed the involvement of JNK-mediated Bcl-2 phosphorylation and degradation, and also the activation of caspase-9 in the apoptosis of both the androgen-dependent and -independent human prostate cancer cells [15]. Bcl-2 and Bcl-XL inhibit apoptosis by regulating the mitochondrial membrane potential, whereas cytochrome *c *release is required for activation of caspase-9 and subsequent activation of caspase-3 [16]. Thus, increased levels of Bcl-2 phosphorylation, caspase-9 and -3 activation appeared to correlate with mitochondrial apoptosis in isochaihulactone-induced LNCaP cell death.

Many microtubule-destabilizing agents are activators of caspase-9, a major key player in mitochondrial apoptotic pathway [17,18]. Microtubule depolymerization agents arrest the cell cycle in G2/M phase by acting through several types of kinases, which lead to phosphorylation cascades, activation of the cyclin B1/cdc2 complex, and the phosphorylation of Bcl-2 [19]. The MAPK inhibitor PD98059 has been shown to partially inhibit isochaihulactone-induced cdc2 phosphorylation, causing G2/M arrest in A549 cells. The activation of NAG-1 expression via ERK1/2 pathway is involved in isochaihulactone-induced G2/M arrest in A549 cells [7,9]. To determine which MAPK family member is involved in the major signaling pathway for isochaihulactone-mediated cell growth inhibition, MAPK inhibitors were used to study the growth inhibition induced by isochaihulactone in LNCaP cells. Only JNK1/2 inhibitor SP600125 significantly decreased the growth inhibition induced by isochaihulactone (Figure 4A), and neither the p38 inhibitor SB203580 nor the ERK1/2 inhibitor PD98059 reversed isochaihulactone-induced growth inhibition. Phosphorylation of JNK kinase was also observed with western blot analysis after isochaihulactone treatment (Figure 4B). In cell cycle analysis, pre-treatment of JNK1/2 inhibitor SP600125 significantly reduces sub-G1 population (Figure 4C). These data suggest that JNK1/2 signaling pathway is involved in isochaihulactone-induce cell death.

Increased NAG-1 expression results in the induction of apoptosis in several cancer cell lines [20,21]. NAG-1 is induced not only by NSAIDs but also by several anti-tumorigenic compounds including dietary compounds, peroxisome proliferator-activated receptor-γ ligands, phytochemicals [16-18], as well as resveratrol, genistein, diallyldisulfide, 5F203, and retinoid 6-[3-(1-adamantyl)-4-hydroxyphenyl]-2-naphthalene carboxylic acid (AHPN) [22-24]. NAG-1 appears to be a key downstream target of EGR-1[9].

In our previously studies, we confirmed the antitumor effect of isochaihulactone [7], and the inhibition of tumor growth that was attributable to NAG-1 protein expression in a nude mice xenograft model [9]. Thus, NAG-1 is an essential factor in the antitumor activity of isochaihulactone. Our current results show that isochaihulactone induced EGR-1 and NAG-1 protein expression in LNCaP cells in a time-dependent manner (Figure 5A). Furthermore, only the JNK1/2 inhibitor SP600125 reduced isochaihulactone-induced NAG-1 protein expression (Figure 5B). These data support that isochaihulactone-induced JNK1/2 activity is critical in regulating NAG-1 expression. In addition, we further confirmed by using siRNA approach that NAG-1 expression has an apoptosis-promoting effect (Figure 5D).

In summary, we found that isochaihulactone increased NAG-1 expression, suggesting that the antitumor effect of isochaihulactone is mediated via this tumor suppressor protein. NAG-1 mRNA is highly expressed in the human prostate epithelium [25], suggesting its role in prostate homeostasis. Despite this, NAG-1 negatively affects LNCaP cell survival [26], and is overexpressed in many tumors including prostate cancer [27,28]. NAG-1 may be like other members of the TGF-β superfamily, acting as a tumor suppressor in the early stages but becoming pro-tumorigenic during the later stages of tumor progression. The effects of NAG-1 appear to be ambiguous, and under different conditions, NAG-1 exhibits either tumorigenic or anti-tumorigenic activity [24]. Epidemiological studies have shown that patients who use NSAIDs for 10-15 years have a reduced risk of developing cancer [29]. NSAIDs inhibit cyclooxygenase-1 (COX-1) and cyclooxygenase-2 (COX-2). Several studies have suggested that the tumorigenic or anti-tumorigenic activity of NAG-1 may be due to the interaction of NAG-1 and cyclooxygenase [21,30,31].

Recent study has revealed a new pathway that Retinoblastoma (RB; encoded by *RB1*) depletion induced unchecked androgen receptor (AR) activity that underpinned therapeutic bypass and tumor progression [32]. The hypo-phosphorylation form of RB suppresses E2F1-mediated transcriptional activation and induces cell cycle arrest. Loss of *RB1 *was observed in most of the castrate-resistant prostate cancer (CRPC), and *AR *as a gene under the control of E2F1, which in turn is stringently regulated by RB. Since hypo-phosphorylation of RB was observed after isochaihulactone treatment in LNCaP cells (data not shown), this might explain why LNCaP is more sensitive to isochaihulactone than the other two androgen-independent prostate cancer cell lines. However, the exact mechanism of these differences needs to be extensively investigated.

## Conclusions

Our current study provides information on the pro-apoptotic and anti-tumorigenic activity of isochaihulactone in human LNCaP prostate cancer cell line. Isochaihulactone downregulated expression of G2/M regulatory proteins including cyclin B1, cdc2, cdc25c, apparently resulting G2/M cell cycle arrest. In addition, isochaihulactone-induced cell death was caspase-dependent and occurred through activations of caspase-9 and caspase-3. The JNK1/2 MAPK signaling pathway and NAG-1 expression were implicated in isochaihulactone-induced cell death. These findings suggest that isochaihulactone has a high therapeutic potential for prostate cancer and should be extensively investigated with *in vivo *studies.

## Competing interests

The authors declare that they have no competing interests.

## Authors' contributions

SCC carried out the most of the experiments and drafted the manuscript. MJW, HHY, YLC, SZL participated in the design and coordination of the study. SPC carried out the statistical analysis. SYH carried out the immunostaining. HJH and CYP conceived of the study, participated in its design and coordination, and drafted the manuscript. All authors read and approved the final manuscript.

## Pre-publication history

The pre-publication history for this paper can be accessed here:

http://www.biomedcentral.com/1471-2407/11/146/prepub
